# Chloroform

**Published:** 1880-03

**Authors:** 


CHLOROFORM.—Dr. A. Hazlewood, in some remarks on this subject in the *Detroit Lancet*, concludes as follows: The points, then, in my argument are briefly: That as an anæsthetic, chloroform has, undoubtedly, many advantages over sulphuric ether.

That in the hands of a competent administrator it is equally safe with sulphuric ether.

That an anæsthetic, when given, should be under the charge of a competent physician or expert; that he should be responsible for that department, as much so as the surgeon operating is in his sphere, and paid a fee therefor commensurate with his ability and time occupied. And to those who may be called upon to perform this duty, I would emphasize the instructions of those writers and experts who have made a study of anæsthesia, to-wit: Quietness in room, and silence of the bystanders; a stomach not too long empty, about four hours since an ordinary meal preferred; loose clothing; recumbent posture (the position on stomach is sometimes dangerous from interference with movements of respiration; sitting up is even worse, tending to produce anæmia of the brain and syncope.) The patient should swallow a small quantity of good distilled liquor before commencing the inhalation.

The inhalation should be at first a large admixture of atmospheric air and gradually increased as the system becomes accustomed, until anæsthesia is perfect, when its further use must be governed by the special circumstances of the case.

The nitrite of amyl and ammonia should be at hand to counteract any bad tendency that may arise. The caution cannot be too often emphasized, never to give an anæsthetic to ladies without a third party being present; nor to expect so pleasant an action when operations upon the genitals or toes are to be made.

